# Commentary: Causal associations between inflammation, cardiometabolic markers and schizophrenia: the known unknowns

**DOI:** 10.1093/ije/dyz201

**Published:** 2019-09-28

**Authors:** Golam M Khandaker

**Affiliations:** 1 Department of Psychiatry, University of Cambridge School of Clinical Medicine, Cambridge, UK; 2 Cambridgeshire and Peterborough NHS Foundation Trust, Cambridge, UK; 3 National Institute for Health Research Cambridge Biomedical Research Centre, Cambridge, UK

Observational studies have extensively documented immune and metabolic dysfunctions in schizophrenia,^1^^,^^2^ an archetypal psychotic disorder characterized by difficulties with, primarily, perception (e.g. hallucinations, delusions) and cognition (e.g. poor attention, memory). With lifetime prevalence of about 1%, schizophrenia typically manifests during the second and third decades of life, has a chronic relapsing remitting course and is thought to be linked with altered neurodevelopment.^3^ Pathophysiological explanation and drug treatment for the illness are predicated on dopaminergic overactivity in the mesolimbic pathway and underactivity in the mesocortical pathway, but this is far from the full picture.^4^ Dopamine overactivity is not present in all patients with schizophrenia, and about a third of patients do not respond to anti-dopamine antipsychotic drugs currently used to treat this illness,^5^ suggesting that other mechanisms are involved.

Emerging evidence suggests that inflammation could be among the causes of schizophrenia, rather than simply being a consequence of illness (reverse causality) or a result of confounding by lifestyle and other factors.^1^^,^^6^ Meta-analysis of cross-sectional studies confirms elevated concentrations of C-reactive protein (CRP), an acute phase protein and archetypal inflammatory marker, and inflammatory cytokines such as interleukin 6 (IL-6) in peripheral blood in patients with schizophrenia compared with controls, which tend to normalize after recovery but continue in treatment-resistant patients.^7–9^ Population-based longitudinal studies have reported associations of elevated concentrations of CRP, IL-6 and erythrocyte sedimentation rate (ESR) in childhood, adolescence or young adulthood with risk of psychotic symptoms or diagnosis of schizophrenia subsequently in adulthood.^10–12^ These studies go some way toward addressing reverse causality. Furthermore, they have accounted for several potential confounders, but residual confounding still may explain the association between inflammatory markers and psychotic disorders.

Cardiovascular and metabolic diseases are among the leading contributors to reduced life expectancy and increased mortality in people with schizophrenia and related psychotic disorders.^13^ Increased risks for these physical illnesses in people with schizophrenia are commonly attributed to the diabetogenic effect of antipsychotic drugs, sedentary lifestyle, poor diet, smoking and alcohol use. However, it is clear that side effects of antipsychotic drugs are unlikely to fully account for increased metabolic illness in patients with schizophrenia. Increased risk of type two diabetes in these patients was reported in studies from the asylum era before the introduction of modern antipsychotic drugs.^14^ Recent studies have reported disruption of glucose and insulin homoeostasis in antipsychotic-naïve first-episode psychosis patients^2^ and in young people with psychotic experiences.^15^ But, of course, other confounders may still be at play.

Mendelian randomization (MR) analysis has provided an innovative and useful way of addressing residual confounding;^16^ a key issue for observational studies. A study by Lin and colleagues in this issue of the *International Journal of Epidemiology* has applied novel MR approaches to test causality of association for schizophrenia with CRP and a range of metabolic markers.^17^ This study has many strengths. While there are a number of MR studies testing the association of schizophrenia with individual inflammatory markers,^18–20^ and with certain cardiometabolic risk factors,^21^^,^^22^ this study included a broad range of cardiometabolic markers along with CRP. The study relied on large, up-to-date GWAS for summary statistics for gene–exposure, gene–outcome associations. Particular methods for MR analysis, such as generalized summary-data-based MR (GSMR) and multi-variable MR (MVMR), are innovative and appropriate given the large list of exposures examined. In addition, examination of pleiotropy, heterogeneity and measurement error using various sensitivity analyses increases confidence in the findings.

Using bidirectional MR analysis, the authors report that CRP could be causally linked with schizophrenia (elevated CRP level decreases risk), but schizophrenia is unlikely to be causally linked with CRP concentrations/activity.^17^ These findings are consistent with a previous MR study that also reported a protective effect of higher CRP levels for schizophrenia,^18^ but are distinct from MR studies of depression, where higher CRP levels were reported to increase the risk of illness.^23^ The MR findings showing a protective effect of CRP on schizophrenia risk contrast with a number of previous observations regarding the immune–schizophrenia link: (i) meta-analyses of cross-sectional studies reporting increased CRP levels in patients with schizophrenia compared with controls,^7–9^ and that about a quarter of patients with schizophrenia have elevated CRP levels;^24^ (ii) longitudinal studies reporting an association between elevated inflammatory markers (e.g. IL-6, CRP, ESR) at baseline and higher risk of schizophrenia and related psychotic disorders subsequently at follow-up;^10–12^ (iii) higher levels of inflammatory cytokines, such as IL-6, at presentation are associated with poor treatment response in patients with psychotic disorders.^25^ So how do we reconcile this contrast between genetic and observational epidemiological findings regarding the potential role of CRP/inflammation in the pathogenesis of schizophrenia?

Clearly, a potential role of inflammation in schizophrenia pathogenesis is not as straightforward as once thought, given that protective effects of high CRP levels have been reported from MR analysis by Hartwig *et al.*^18^ and replicated here by Lin *et al*.^17^ As noted by Hartwig and colleagues,^18^ low CRP could perhaps increase schizophrenia risk by increasing the risk of infection. Indeed, there is extensive literature linking adult schizophrenia with increased prevalence of infection and autoimmunity in adulthood,^26^ as well as infection during prenatal life and childhood.^27–30^ Therefore, it is possible that inflammatory overactivity seen in adult schizophrenia patients is in fact influenced by genetically driven deficits in CRP levels/activity in early life that predispose these individuals to infection. This hypothesis warrants investigation using prospective cohort, immunophenotyping and genetic approaches. However, even if this were true, inflammation could still be a valid treatment target or marker of treatment response in patients with schizophrenia. Whether this is indeed the case requires testing using experimental medicine approaches.

It is worth noting that genetic predisposition for schizophrenia, as indexed by polygenic risk score, is not associated with risk of infection,^31^ suggesting that increased infection risk in schizophrenia patients could be due to environmental factors. Consistent with this idea, a recent study using co-relative control analysis of a large Swedish general population-based cohort has reported that the association between childhood infection and schizophrenia could be attributed to unique environmental factors, rather than shared genetic or shared environmental factors.^32^ The study shows that infection–psychosis associations are similar in the general population and in full-sibling pairs discordant for exposure.^32^ What these unique environmental factors are, and how they influence immune function and, consequently, schizophrenia risk are open questions for the field.

Another possibility is that the nature association (i.e. protective vs harmful) between low-grade inflammation, as measured by circulating inflammatory markers, and schizophrenia changes in different stages of development. A longitudinal study from Sweden reported an association between low acute phase protein levels after birth, measured in blood spots, and risk of psychotic disorders in adulthood.^33^ However, using prospective data from the UK,^11^ Finland^12^ and Sweden,^10^ we have reported that inflammatory overactivity during childhood, adolescence and young adulthood (increased levels of IL-6 at age 9, CRP at age 15 and ESR at age 18) are associated with increased risk of psychotic symptoms or diagnosis of schizophrenia subsequently in adulthood. Whether, indeed, the nature of association between schizophrenia and inflammatory markers varies depending on stage of development requires examination using repeat measurements of CRP and other inflammatory markers from the same individuals over time. Such studies are currently lacking.

Lin *et al*. also report potential causal associations for three further biomarkers: triglycerides, citrate and lactate (higher triglycerides increase risk of schizophrenia, but higher citrate and lactate decrease risk).^17^ Triglycerides were reported to be potentially causally associated with depression in a previous MR study based on UK Biobank (UKB) data.^23^ The association of triglycerides with depression is unlikely to be driven by central obesity, because in the UKB study, body mass index (BMI) or waist-to-hip ratio (WHR) were are not causally linked with depression.^23^ In future, MR studies of cardiometabolic biomarkers in schizophrenia should also include BMI, WHR, etc. to disentangle whether any associations for triglycerides are driven by central obesity. Nevertheless, there seems to be a consistent signal for an association between triglycerides and risks of depression and schizophrenia. In future, studies should examine mechanisms through which alterations in triglycerides contribute to risk of major neuropsychiatric illness to elucidate potential targets for treatment and prevention.

Another important avenue for research in future would be to examine the interplay between immune and metabolic dysfunction in relation to schizophrenia risk. This was not tackled by Lin *et al*., who analysed CRP and metabolic markers separately.^17^ Whereas they included a comprehensive array of metabolic markers and CRP, future studies should consider including other inflammatory markers, e.g. IL-6 and other cytokines, that have been consistently linked with schizophrenia risk in observational studies.^7–9^ There is evidence that inflammation can contribute to metabolic dysfunction. Inflammation leads to changes in lipid metabolism, including increased triglycerides, decreased HDL cholesterol^34^ and insulin resistance.^35^ Anti-inflammatory treatment that inhibits IL-6 also inhibits triglycerides.^36^ Using the MR approach, a study has reported that inflammation could be a shared mechanism for depression and cardiovascular disease (CVD).^23^ It is possible that inflammation is also a shared mechanism for metabolic dysfunction and schizophrenia. This hypothesis needs testing. In future, MVMR or MR analyses based on genetic variants with known biological action may help to disentangle shared and unique contributions of immune and metabolic factors in schizophrenia pathogenesis. Such endeavours to identify shared mechanisms for commonly comorbid major psychiatric and physical illnesses of adult life, such as schizophrenia, depression, type two diabetes and CVD, could open up novel avenues for treatment and prevention of these conditions, which, together, contribute to significant health-related morbidity and mortality worldwide.

## Funding

G.M.K. acknowledges funding support from the Wellcome Trust (Intermediate Clinical Fellowship; grant code: 201486/Z/16/Z), the MQ: Transforming Mental Health (Data Science Award; grant code: MQDS17/40), and the Medical Research Council (MICA: Mental Health Data Pathfinder; grant code: MC_PC_17213). 
