# Corrigendum to: Commentary: Causal associations between inflammation, cardiometabolic markers and schizophrenia: the known unknowns

**DOI:** 10.1093/ije/dyz226

**Published:** 2019-10-31

**Authors:** Golam M Khandaker

First published online: 28 September 2019, *Int J Epidemiol* 2019; 48: 1516–18. doi: https://doi.org/10.1093/ije/dyz201

The originally published version of this article contained a typographic error in the title; this has now been corrected.

